# Chemical camouflage: a key process in shaping an ant-treehopper and fig-fig wasp mutualistic network

**DOI:** 10.1038/s41598-018-20310-7

**Published:** 2018-01-30

**Authors:** Bo Wang, Min Lu, James M. Cook, Da-Rong Yang, Derek W. Dunn, Rui-Wu Wang

**Affiliations:** 1 Key Laboratory of Tropical Forest Ecology, Xishuangbanna Tropical Botanical Garden, Chinese Academy of Sciences, Menglun, 666303 China; 2 Center for Ecological and Environmental Sciences, Northwestern Polytechnical University, Xi’an, 710072 China; 3 State Key Laboratory of Integrated Management of Pest Insects and Rodents, Institute of Zoology, Chinese Academy of Sciences, Beijing, 100101 China; 4 Hawkesbury Institute for the Environment, University of Western Sydney, Hawkesbury Campus, Locked Bag 1797, Penrith, 2751 NSW Australia; 5 College of Life Sciences, Northwest University, Xi’an, Shaanxi 710069 China

## Abstract

Different types of mutualisms may interact, co-evolve and form complex networks of interdependences, but how species interact in networks of a mutualistic community and maintain its stability remains unclear. In a mutualistic network between treehoppers-weaver ants and fig-pollinating wasps, we found that the cuticular hydrocarbons of the treehoppers are more similar to the surface chemical profiles of fig inflorescence branches (FIB) than the cuticular hydrocarbons of the fig wasps. Behavioral assays showed that the cuticular hydrocarbons from both treehoppers and FIBs reduce the propensity of weaver ants to attack treehoppers even in the absence of honeydew rewards, suggesting that chemical camouflage helps enforce the mutualism between weaver ants and treehoppers. High levels of weaver ant and treehopper abundances help maintain the dominance of pollinating fig wasps in the fig wasp community and also increase fig seed production, as a result of discriminative predation and disturbance by weaver ants of ovipositing non-pollinating fig wasps (NPFWs). Ants therefore help preserve this fig-pollinating wasp mutualism from over exploitation by NPFWs. Our results imply that in this mutualistic network chemical camouflage plays a decisive role in regulating the behavior of a key species and indirectly shaping the architecture of complex arthropod-plant interactions.

## Introduction

Mutualism is a common and widespread ecological interaction^1^. The partner species of a single mutualism often interact with other community members to form a complex interacting network, which is regarded as the ‘architecture of biodiversity’^2,3^. Within a network, a species may be the mutualistic partner of one species, but also a predator, competitor, or prey item of other species^4,5^. Such species may have an extraordinary high degree of interaction toward other species and might result in significant changes in the wider species interaction networks^5^. However, the role such high interplay species have in the network and the underlying mechanisms that enable stability of such networks are both poorly understood.

Interactions between ant (Hymenoptera: Formicidae) and hemipteran insect species (e.g., leafhopper, treehopper) are abundant and widespread in arthropod food webs and they commonly form a food-for-protection mutualism^6^. Besides, ants are generally polyphagous and prey on other insects, including herbivores and pollinators, which can potentially shape plant-pollinator and plant-herbivore interactions^7,8^. Therefore, mutualisms between ant and hemipteran insects are considered the ‘keystone interactions’ in arthropod communities and their host plants^8^. Moreover, ants have a high interplay with other arthropod species and may directly and indirectly shape arthropod community structure^9^. The presence of the ants reduces hemipteran predation/parasitism rates and in return the hemipterans provide the ants a honeydew food reward^10^. It was long believed that hemipteran honeydew can suppress ant aggression to other organisms and allow the ants to start tending activities^11^. However, when alternative food resources are limited, the ants themselves may prey upon their hemipteran partners^12^, which suggests that the benefits associated with honeydew are not always enough to maintain the mutualism.

Chemical signals and their perception are important in ant foraging and inter/intra specific recognition, and have been frequently reported to reduce ant aggression and contribute to mutualism stability^13–15^. It has been frequently reported that chemical signals can manipulate ant behavior to favor many insect mutualists, making them less likely to be preyed on by the ants^14,16^. However, studies on the role of chemical signals in maintaining the stability of mutualisms and regulation predation behavior of one species are uncommon.

Weaver ants (*Oecophylla smaragdina*) often build leaf nests on fig trees^4^. These ants are voracious predators of other small insects^17^, including the pollinating and non-pollinating fig wasps (NPFWs) that can only use the flowers within the inflorescences of fig trees (syconia, colloquially ‘figs’) as larval nurseries^7^. The presence of *O*. *smaragdina* can help prevent some NPFWs from out-competing pollinators by selectively preying upon those species that are independent gall makers^4^, and may also reduce the likelihood of the larvae of pollinating fig wasps from being attacked by NPFW parasitoids^18^. The aggressive, predatory behavior of *O*. *smaragdina* towards some NPFWs differs from that often directed at honeydew producing hemipterans, which concurs with the classical food-for-protection ant-hemipteran model (Digital appendices 1 and 2).

It has been frequently reported that chemical resemblance (mimicry or camouflage) can manipulate ant behavior to favour many species of phytophagous insect mutualists, making them less likely to be preyed on by the ants^14,16^. Here, we used a weaver ant-treehopper mutualism associated with the fig tree species *Ficus racemosa* in southwestern China to test if the behaviors of the ants are affected by chemical signals given by treehopper mutualisms. We hypothesized that chemical signals produced by the treehoppers may manipulate the behavior of the ants in their favor and thus help maintain the stability of this system. We attempted to answer five questions (1) Does *O*. *smaragdina* have an intimate relationship with a species of treehopper? (2) Do the chemical profiles of the branches of *F*. *racemosa*, and the different species of fig wasps and treehoppers, and *O*. *smaragdina*, associated with *F*. *racemosa* at our study site differ or are they similar? (3) Is *O*. *smaragdina* attracted by olfaction to any of these chemical signals? (4) Do the chemical profiles of any members of this mutualism network reduce the likelihood of treehoppers being preyed on by ants in the absence of any honeydew reward? (5) Do different *O*. *smaragdina* treehopper abundances affect fig-fig wasp community structure? Our results clarify whether chemical resemblance (mimicry or camouflage) exists in an ant-treehopper mutualism, if chemical mimicry or camouflage has the potential to indirectly contribute to stability of an ant-treehopper mutualism, and the potential ecological consequences of an ant-treehopper mutualism for a wider mutualism network.

## Results

### The association between ant and Tricentrus abundances

**Figure 1:**
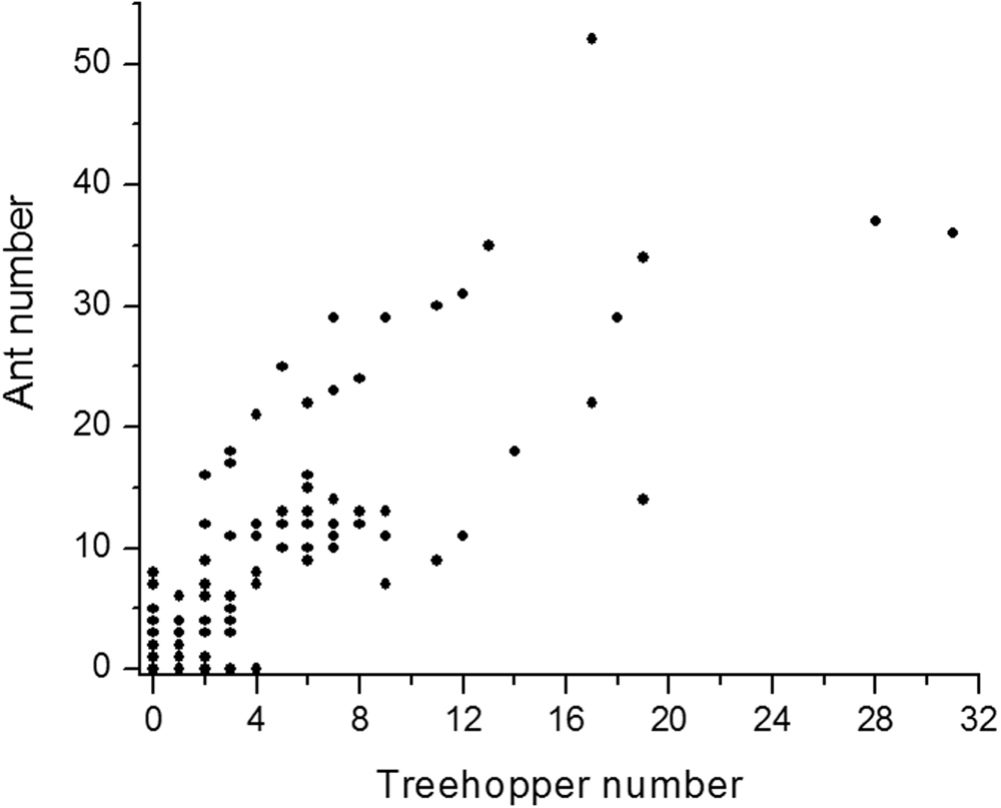
The relationship between *Oecophylla smaragdina* and treehopper (*Tricentrus* sp.) abundances on *Ficus racemosa*.

Each cluster averaged 10.24 ± 7.34 (mean ± SD) figs. For each fig cluster, there were 9.23 ± 10.48 ants and 4.38 ± 5.7 *Tricentrus*. Ant and *Tricentrus* abundances were significantly positively correlated (Pearson’s correlation r = 0.81, *P* < 0.01, N = 113, Fig. 1).

### Chemical profile analysis

**Figure 2:**
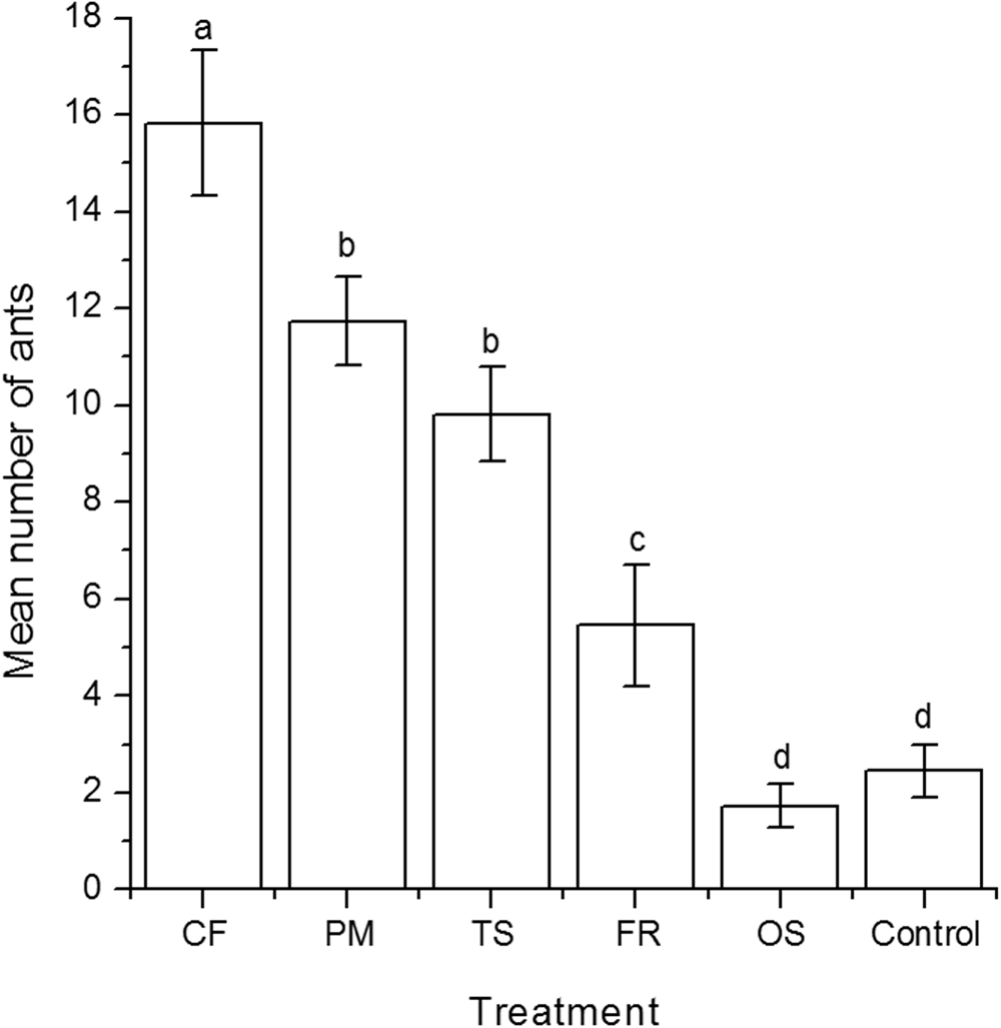
Non-metric multidimensional scaling analysis based on cuticular chemical components of *Oecophylla smaragdina* (OS), *Ceratosolen fusciceps* (CF), *Platyneura mayri* (PM), and *Tricentrus* sp. (TS), and surface chemicals of *Ficus racemosa* branches (FR).

We identified 47 chemicals from the cuticles of ants, wasps, *Tricentrus*, and the branch surfaces of *F*. *racemosa* (supplementary dataset 1). Chemicals were categorized to either alcohols (8), alkanes (12), aldehydes (2), alkenes (11), carboxylic acids (5), esters (4), ethers (1), ketones (1), olefine ketones (1), and phenols (2). *F*. *racemosa* and *Tricentrus* shared 8 chemicals. NMDS comparisons of the chemical profiles among ants, wasps, *Tricentrus*, and *F*. *racemosa* branches are shown in Fig. 2 (stress value = 0.04, R^2^ = 0.998), in which *Tricentrus* have the least NMDS distance to *F*. *racemosa* branches. In the cluster analysis, the cuticular hydrocarbons of *Tricentrus* and the surface chemical profile of *F*. *racemosa* inflorescence branches were most similar, with the next most similar profile being the cuticular chemicals of *C*. *fusciceps*.

### Attractiveness to ants of chemicals of conspecifics, fig wasps, Tricentrus, and F. racemosa

**Figure 3:**
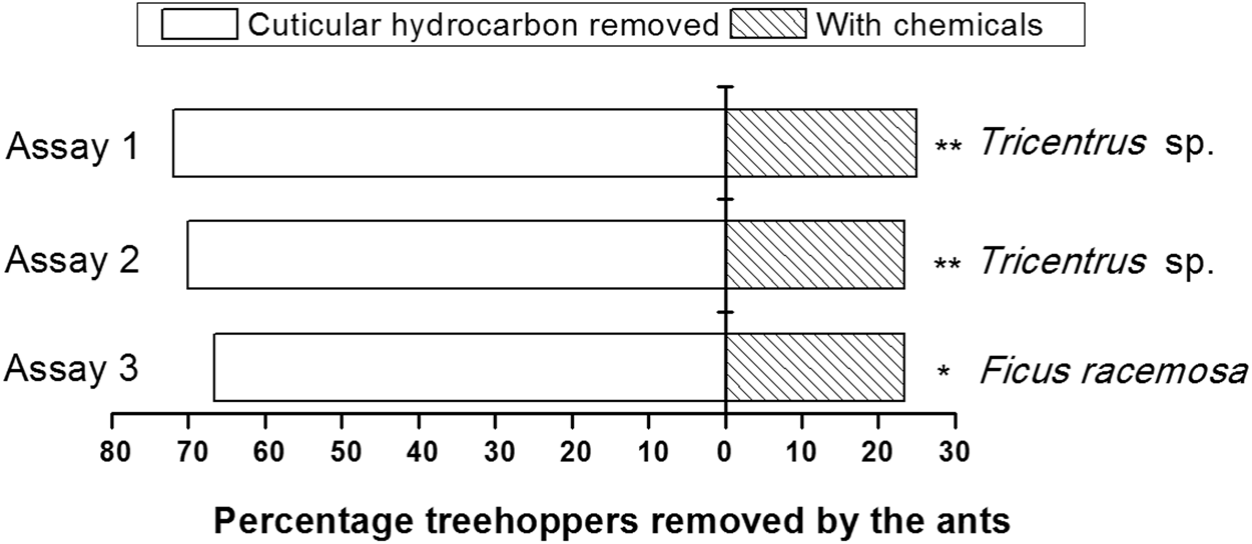
The numbers of *Oecophylla smaragdina* aggregated on chemicals from inflorescences branches of *Ficus racemosa* (FR), or the cuticles of *Ceratosolen fusciceps* (CF), *Platyneura mayri* (PM), *Tricentrus* sp. (TS), *Oecophylla smaragdina* (OS), and controls (hexane). The pairwise comparisons between chemicals using Tukey’s HSD (honest significant difference).

Ant attractions to cuticular chemicals from pollinating fig wasps, non-pollinating fig wasp, *Tricentrus*, fig branches, and conspecifics are differs significantly (GLMM, χ^2^ = 233.91, P < 0.0001). Cuticular chemicals from pollinating fig wasps are most attractive to ants (15.82 ± 4.98) followed by non-pollinating fig wasp (11.73 ± 3.04), treehoppers (9.82 ± 3.28), fig branches (5.45 ± 4.16), controls (2.45 ± 1.81), and conspecifics (1.73 ± 1.49) (Fig. 3).

### The role of cuticular hydrocarbons in preventing treehoppers from attacking ants

**Figure 4:**
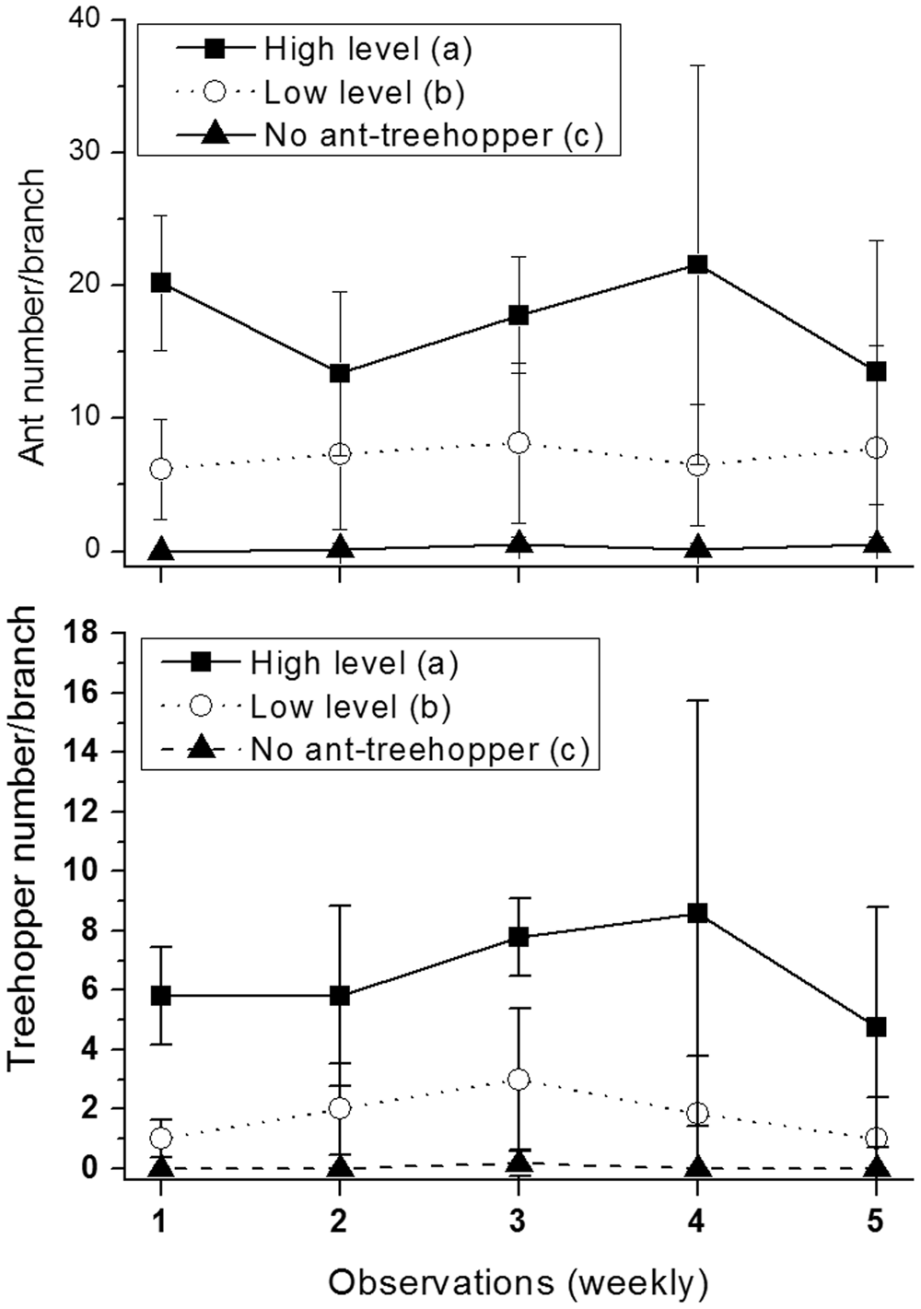
Percent of *Oecophylla smaragdina* that selected treehopper (*Tricentrus* sp.) nymphs with or without cuticular hydrocarbons from *Tricentrus* sp. or *Ficus racemosa* twigs. Chi-square test, *P < 0.05, **P < 0.01.

In the first behavioral assay, the ants removed significantly more *Tricentrus* without cuticular hydrocarbons (72%) than *Tricentrus* that had intact cuticular hydrocarbons (25%) (χ^2^ = 7.26, *P* < 0.01, n = 32). The ants failed to remove any of the two *Tricentrus* in only one replicate (3%). In our second assay, ants preyed most on *Tricentrus* that had their cuticular hydrocarbons removed (70%) relative to controls (23%) (χ^2^ = 7.00, *P* < 0.01, n = 30) (Fig. 4). In two replicates (7%), no *Tricentrus* were preyed on by ants during 15 minutes. In the third assay, significantly fewer *Tricentrus* that had their cuticular hydrocarbons replaced with the branch-surface chemicals from *F*. *racemosa* (23%) suffered ant predation, than those that had their cuticular hydrocarbons removed (67%) (χ^2^ = 6.26, *P* < 0.05, n = 30). In three replicates (10%) ants failed to remove any *Tricentrus*.

### The effects of the ant-treehopper mutualism on the fig wasp community

**Figure 5:**
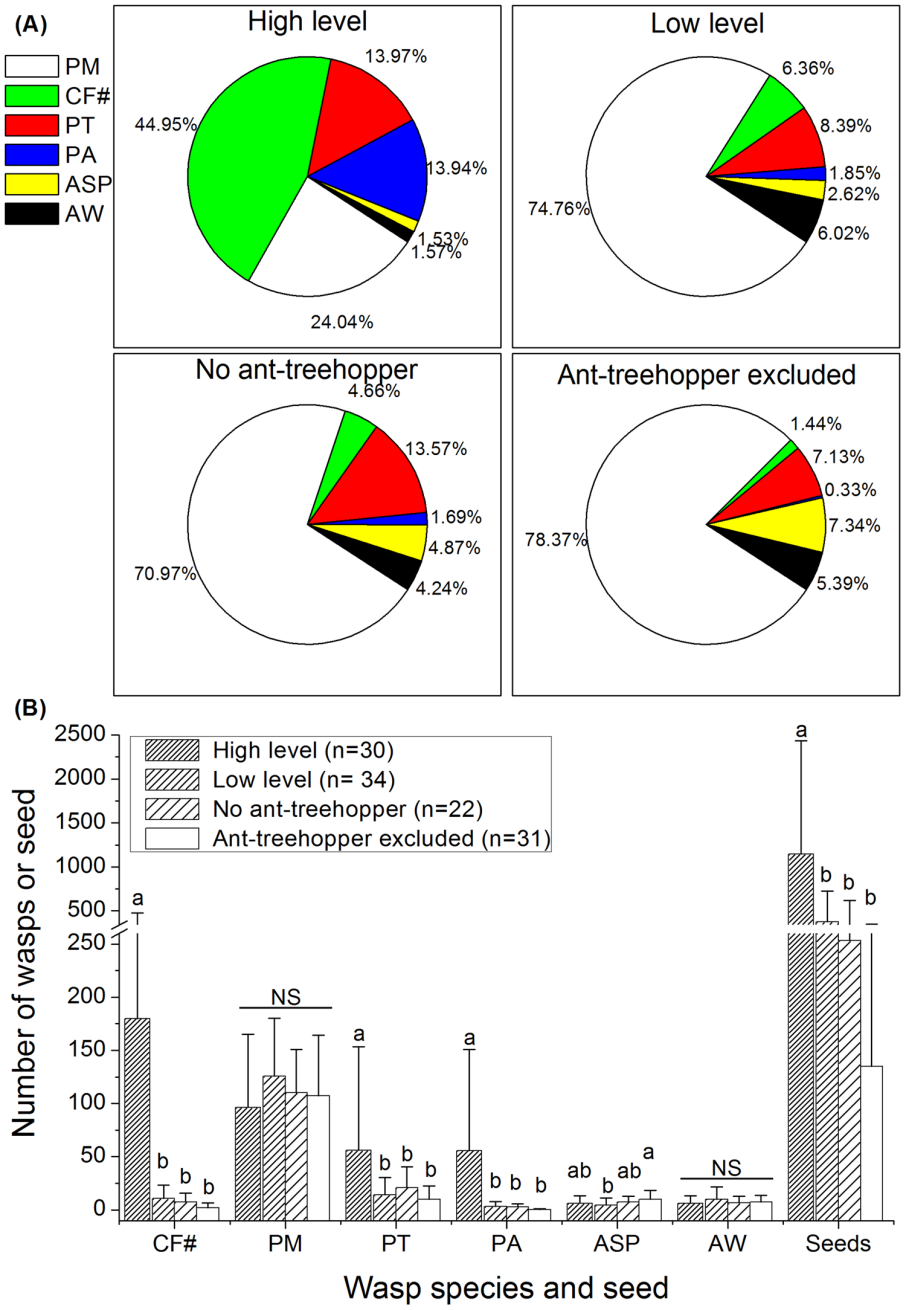
The number of wasps from branches with different levels of ant-treehopper abundances: (**A**) species composition, and (**B**) comparisons of wasp and seed numbers among different levels of ant-treehopper abundance. Columns and bars show mean ± SD. Different letters indicate significant differences between groups of ant-treehopper abundance levels, by S-N-K multiple pair-wise comparisons. NS = not significant. CF = *Ceratosolen fusciceps*; PM = *Platyneura mayri*; PT = *Platyneura testacea*; PA = *Platyneura agraensis*; ASP = *Apocrypta* sp.; AW = *Apocrypta westwoodi*. # represent the pollinating fig wasps, others are non-pollinating fig wasps.

The fig wasp community structure significantly different among levels of ant-treehopper abundance (ANOSIM, R = 0.06, *P* < 0.01). For branches with many ants, pollinating fig wasps were the dominant species (44.95%), followed by *P*. *mayri* (24.04%), and then *P*. *testacea* (13.97%) and *P*. *agraensis* (13.94%). Individuals of the remaining two wasp species accounted for <5% of all species present at the time of sampling. On branches with few or no ants, *P*. *mayri* was the dominant species (>70% of all wasps present), followed by *P*. *testacea* (8.39% with few ants present, 13.57% with no ants, and 7.13% when ants were excluded). The proportion of pollinating wasps decreased according to reduced levels of ant-treehopper abundance; 44.95% with many ants, 6.36% with few ants, 4.66% with no ants, 1.44% when ants were excluded. No clear patterns were present for the remaining three species of NPFWs (Fig. 5A).

The abundance of ant-treehopper mutualists significantly affected fig seed production (*F*_3,112_ = 8.15, *P* < 0.001) and the number of pollinating fig wasps (*F*_3,112_ = 7.41, *P* < 0.001). Furthermore, the abundances of three species of NPFWs *P*. *agraensis* (*F*_3,112_ = 7.14, *P* < 0.001), *A*. sp. (*F*_3,112_ = 3.01, *P* < 0.05), and *P*. *testacea* (*F*_3,112_ = 3.40, *P* < 0.05) were also significantly affected by the abundance of ant-treehopper mutualists (Fig. 5B). However, the abundances of *A*. *westwoodi* (*F*_3,112_ = 1.90, *P* = 0.13), and *P*. *mayri* (*F*_3,112_ = 1.24, *P* = 0.30) were not affected (Fig. 5B).

## Discussion

Ants can play important roles in terrestrial ecosystems because they often interact closely with multiple species. Ant behavior is sensitive to chemical signals from con-specifics, symbionts, prey, predators, and the wider environment in which they forage and build nests^19^. Behavior responses of ants to one or more of these factors may indirectly affect the interspecific interactions with other species of a community of which the ants are a component. Our results show that the cuticular hydrocarbons of treehoppers on one hand attract mutualistic weaver ants, but on the other hand help protect the treehoppers from ant predation even in the absence of honeydew rewards. Our data suggest that this reflects the similarity between treehopper cuticular hydrocarbon profiles and fig tree inflorescence branch surface chemicals. Cuticular chemicals from treehoppers and fig inflorescences both attract weaver ants, which may reflect a long history of co-evolution among the three species. Further behavioral experiments provide evidence that chemical extracts from both treehoppers and fig inflorescence branches can independently protect treehopper nymphs from ant predation when honeydew is absent. In the field, an abundance of ant-treehopper mutualists has positive effects on the production of pollinating fig wasps and seeds, probably via weaver ant predation on the NPFWs that oviposit on the outer surface of figs^4^. Thus, weaver ants probably have an important function in this mutualistic network.

One organism can chemically resemble another in either of two ways: (1) mimicry and (2) camouflage^20^. Camouflage is the simulation of the general background, which is a common strategy to reduce ant predation in phytophagous insects^21^. In the field, *O*. *smaragdina* often forages along the small inflorescence branches of *F*. *racemosa* and shows “tending behavior” to both *Tricentrus* nymphs and the bracts of small fig syconia on the inflorescence branches. We found no evidence that *O*. *smaragdina* can distinguish between *Tricentrus* and the inflorescence branches of host fig trees, because they failed to consistently find the *Tricentrus* and also demonstrated “tending behavior” to *F*. *racemosa* branches. Because fig inflorescence branches are a background object for *O*. *smaragdina*, we suggest that chemical camouflage explains to a large degree the behaviour of *O*. *smaragdina* towards *Tricentrus* nymphs.

Although the surface chemicals of *Tricentrus* and small fig tree branches have the smallest pair-wise NMDS distance for all the five species we measured, the difference of in overall chemical composition between *Tricentrus* and fig tree branches is obvious. However, only a subset of chemicals is responsible for insect inter/intra specific recognition and similarity in these info-chemicals will achieve chemical camouflage^22^. Moreover, we also demonstrated experimentally that *Tricentrus* cuticular hydrocarbons and surface chemicals from fig trees can independently protect *Tricentrus* from ant attack in the absence of any honeydew rewards, and therefore reduce punishment by ants for non-cooperative behavior. However, the precise mechanism for this camouflage remains unknown. Whether *Tricentrus* treehoppers sequester compounds from the plants or biosynthesize them independently requires further investigation, as does the function of the individual chemicals responsible for affecting ant behavior. Some chemicals present in both treehoppers and fig inflorescence branches may function as camouflage chemicals but others may attract ants, and both quantitative and qualitative reactions may be involved.

In general, mutualisms between ants and hemipterans are not species specific^12^. Ants can learn to associate honeydew production with the chemical profiles of hemipteran (aphid) cuticles through chemically-mediated associative learning^23^. Such an association can be established in short time periods in the laboratory, with some ant species even showing tending behavior towards dead hemipterans which fail to respond to any stimuli from the antennae of ants, as long as the hemipteran cuticular chemical profiles remain intact^13^. We found that *O*. *smaragdina* showed tending behavior to filter papers treated with chemicals from either *Tricentrus* or fig inflorescence branches, which indicated that chemical signals can help enforce the ants to behave cooperatively towards their treehopper mutualists. Our data therefore suggest an association between treehopper chemical cues and the production of honeydew, which may reduce any costs of association incurred by the treehoppers. Ant associative learning may contribute to the persistence of this mutualism^24^, with chemical camouflage being used by treehoppers to avoid costs inflicted by ants.

One factor that contributes to stability in mutualisms is actively rewarding cooperation and/or punishing less mutualistic behavior^25^. In some cases, sanctions against less mutualistic partners occur^25,26^, although the implementation of sanctions may be costly and mutualistic partners that provide reduced benefits to hosts still provide net benefits^26^. In the weaver ant-treehopper mutualism, ants may prey on hemiptera when food resources are limited^12^, which may act as an indirect sanction by ants to less cooperative hemipterans. However, predation on hemiptera may result in a net cost to the ants due to less food in the long term, which may be detrimental to the mutualism. Sakata^11^ reported that if aphids provided potentially aggressive ants honeydew, and the honeydew was consumed, predation rates were lower than when the aphids failed to provide honeydew. Individual treehoppers might temporarily be unable to pay honeydew to weaver ant partners, because of a lack of resources, time constraints, or poor physical condition. Chemical camouflage may reduce any errors in the application of sanctions by ants against non- or less cooperative treehoppers, and may thus be important in helping maintain the stability of this system. Although a possible consequence of chemically mediated behavioral manipulation in this system is to reduce the effectiveness of resource/service exchange processes, it may also underpin the mutualism between weaver ants and treehoppers. Contrary to previous work^27^, we suggest that chemical camouflage may not only be a consequence, but also a possible contributory factor enabling a stable inter-specific mutualism.

**Figure 6:**
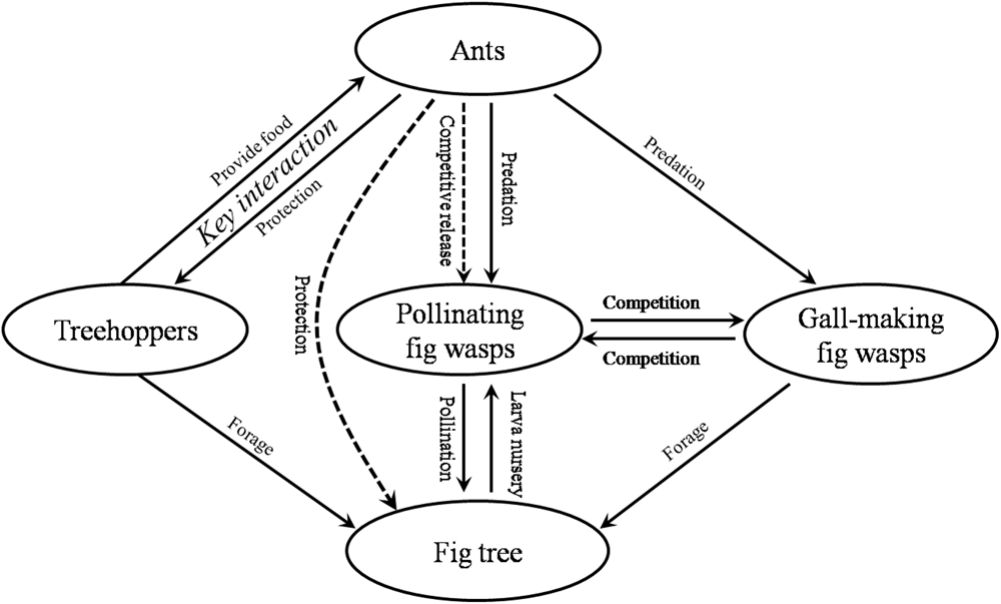
A schematic diagram of the mutualism network of ants, treehoppers, fig wasps, and *Ficus racemosa*. Chemical signals helps maintain the mutually beneficial interaction between ants and treehoppers, and ant presence facilitates reproductive success of both fig-pollinator mutualists. Solid lines represent direct effects, dashed lines represent indirect effects.

Furthermore, *O*. *smaragdina* has important ecological interactions with additional invertebrate species that are associated with fig trees. For instance, *O*. *smaragdina* reduces the oviposition rates of some non-pollinating fig wasps (NPFWs), which attack figs from their outer surface. When their long ovipositors are fully inserted into a fig, NPFWs are vulnerable to ant attack (See Digital appendices, Digital appendix 2^28,29^). Because some NPFW species are competitors or parasitoids of the pollinators, and may also reduce seed production, ant attacks thus indirectly promote the mutualism between fig trees and their pollinating wasps^4^. These ants therefore help maintain two mutualistic relationships: (1) with *Tricentrus* by their tending and predatory behaviors, and (2) between the fig tree and its pollinating wasp by preying on some NPFW species whose larvae compete with pollinator larvae within their host fig^4^. Chemical signaling is thus crucial in regulating ant behaviour and shaping this mutualistic network (Fig. 6).

## Materials and Methods

### Location and study system

The study was carried out in the Xishuangbanna Tropical Botanic Garden (21°41′N, 101°25′E), Yunnan province, southwest China. *Ficus racemosa* is a monoecious fig species pollinated by *Ceratosolen fusciceps* Mayr. It produces large crops of large figs (syconia: approx. 40 mm in diameter when mature), which are borne on small branches that grow from the trunk and larger branches. At Xishuangbanna, five non-pollinating fig wasp species reproduce in the syconia of *F*. *racemosa*; three species of *Platyneura* (*P*. *mayri*, *P*. *testacea*, and *P*. *agraensis*) and two of *Apocrypta* (*Apocrypta* sp. and *A*. *westwoodi*)^30^. The syconia have five distinct developmental stages^31^: pre-receptive, receptive to pollination, inter-floral, male floral (pollinators mature, acquire pollen and disperse), and seed dispersal, when the figs become attractive to frugivorous seed dispersers. Each wasp species oviposits at a specific developmental stage. The pollinators enter the syconia to oviposit, but all NPFW species associated with *F*. *racemosa* oviposit from the outer fig surface, which makes them vulnerable to predators^4^.

Trophobiotic interactions between *O*. *smaragdina* and honeydew-producing hemipterans on *F*. *racemosa* are common. *Tricentrus* sp. (hereafter *Tricentrus*) is the most common hemipteran associated with *F*. *racemosa* at XTBG. The ants show typical tending behavior (Digital appendix 1), whereby an ant taps the posterior of the abdomen of an individual hemipteran^32^. In response, the *Tricentrus* produces a drop of honeydew at the tip of its abdomen, which the ant then quickly consumes. The shape and size of the *Tricentrus* associated with *F*. *racemosa* is similar to the bracts at the base of an individual fig. On inflorescence branches on which *Tricentrus* are present, ants also frequently tap on the bracts, which suggest that ants may mistake tree branches for treehoppers. In contrast to their tending of honeydew-producing *Tricentrus*, *O*. *smaragdina* is an aggressive predator of other insects closely associated with *F*. *racemosa* trees, including fig-pollinating wasps and NPFWs (Digital appendix 2).

### Behavioral associations between ants and treehoppers

We collected figs of all five different developmental stages from three trees. Each tree was at least 200 m from any other used in the study. For each phase, approximately 30 fig clusters were collected. Random clusters of figs were individually and quickly placed into a fine mesh bag (200 holes per square cm), and then removed from the branch. Each bag, still containing the figs and any insects that were on their surfaces, was put into a freezer for 30 min at −20 °C to anesthetize the insects. All insects were then sorted to order level, and the *Tricentrus* sp. and *O*. *smaragdina* counted.

### The chemical compositions of the external surfaces of F. racemosa branches, *Tricentrus*, *fig wasps*, *and O*. *smaragdina*

We collected crude hydrocarbon extracts from the cuticles of ants, fig wasps, treehoppers, and from the external surfaces of small *F*. *racemosa* twigs. All insects and twigs were collected from the same three *F*. *racemosa* trees as previously used for the behavioural association assessments. Samples were analyzed with a Hewlett-Packard 7890 gas chromatograph-mass spectral (GC-MS) detector.

Figs in the male floral phase (mature figs) were collected and put into a mesh bag to enable the female wasps to be collected after emergence. Thirty wasps per species were immobilized by cooling, placed into a 4 ml Agilent sample vial, and then immersed in 200 µl hexane (chromatographic purity, Merck) for 10 min to dissolve their cuticular hydrocarbons. Only two wasp species were used, the pollinator *C*. *fusciceps* and the galler *P*. *mayri*, because these are the two wasp species associated with *F*. *racemosa* most likely to be preyed upon by *O*. *smaragdina*^4^. All weaver ants and *Tricentrus* sp. were also anesthetized by cooling. For each species, three *O*. *smaragdina* or three *Tricentrus* sp. were immersed in 200 µl hexane in a 4 ml vial for 10 min. Small *F*. *racemosa* inflorescence branch tips (including both the branch and inflorescences buds), each approximately 5 cm long and 0.5 cm in diameter, were removed from a branch. The cut end of each branch was wrapped with cotton wool soaked in tap water to prevent dehydration. In turn, five twigs were sequentially immersed in 4 ml of hexane with each given 10 min to dissolve the surface chemicals. The solvent was then concentrated to 400 µl, using a flow of purified nitrogen. All of the resulting solutions were then filtered with anhydrous sodium sulfate in a glass tube, to remove any water. Each sample (species) had three replicates (three vials).

A sample of each solution (0.2–0.8 µl) was injected into a Hewlett-Packard 7890 gas chromatograph-mass spectral (GC-MS) detector, equipped with an HP-5MS column (5% Phenyl methyl silox, 30 m × 250 µm × 0.25 µm). The temperature program started at 80 °C, and increased at 10 °Cmin-1 until 160 °C, then at 4 °Cmin-1 until 280 °C, and a further 10 °Cmin-1 until 300 °C was reached, and then held for 10 min. The flow of the nitrogen carrier gas in the column was 1.0mlmin-1. Aliquots of extracts were injected splitless at 250 °C. The areas of regularly occurring peaks (those detected at least twice in all of the samples from each source) were included in the subsequent identification and analysis. Chemical profiles were evaluated using the software package Enhanced ChemStation G1701DA Version D.00.01.27. Peaks with the same retention time and mass spectra were considered as the same chemical substances^33^. Each chemical was identified using mass spectra matching with standard mass spectra in the NIST 02 library, and using arithmetic indices (AI) which were calculated based on previously published data on n-alkanes^33^. The relative percentage of each chemical of the total compounds in each sample was calculated according to its percentage peak area of the total peak area of all compounds.

### Are ants attracted to the chemical bouquets of fig wasps, Tricentrus, and F. racemosa?

We measured the behavioral responses of ants to filter papers that had been treated with the crude hydrocarbon extracts from the cuticles of treehoppers, fig wasps, or ants, or the external surfaces of small fig branches. For each of two wasp species (*C*. *fusciceps* and *P*. *mayri*), 60 individuals were placed into a 4 ml sample vial. We then added to each vial 300 µl of hexane, which was left for 10 min to extract the cuticular hydrocarbons of the insects. For the plant extract, five small tree branches were rinsed sequentially with 4 ml hexane for 10 min each, and the resulting solution concentrated to 400 µl. For the ant and leafhopper extracts, three individuals per species were put into a 4 ml sample vial. We then added 300 µl hexane, which was left for 10 min. All insects and branches were removed from the vials after extraction.

For the behavioural assays, five microlitres of the crude extracts from the wasps, ants, treehoppers, or inflorescence branches were each placed onto one 5 mm × 5 mm square of filter paper. All papers were left to dry for 1 min. The five filter papers were then stapled to one large *F*. *racemosa* branch. Specific positions of filter papers with chemicals were randomly assigned in order for each replications by pseudorandom number. The number of ants that each made contact with each paper was recorded every minute for 15 minutes and all counts were summed for total scores.

A preliminary experiment showed that a hexane-only treated filter paper had no significant effect on ant behaviour compared with a dry filter paper. In a dual choice assay, ants simply removed with equal likelihood dry or hexane treated filter papers (60 replicates). However, in the main experiment the ants were more likely to carry the filter papers with fig wasp cues back to their nest. We thus used only a dry filter paper of equal size to the treated papers as a control in each replicate. The assay had 11 replicates on three different *F*. *racemosa* branches. Each branch was on a separate tree, with each tree having at least three replicates.

### The role of cuticular hydrocarbons in protecting treehoppers from ant attack

To measure the effects of cuticular hydrocarbons from *Tricentrus* and surface chemicals of fig inflorescence branches on the behavior of *O*. *smaragdina*, we performed three dual choice field experiments. Each experiment had at least 30 replicates spread over three different *F*. *racemosa* trees. Several treehoppers (4th or 5th instar nymphs) were killed by being placed into a freezer at −80 °C for 5 min. Each nymph was then randomly assigned to one of two groups.

The aim of our first assay was to test if the cuticular chemical profiles of *Tricentrus* sp. offer protection from ant predation. For controls, nymphs were un-manipulated; for a treatment group, each nymph was rinsed in 100 μl hexane for 10 min to remove its cuticular hydrocarbons and then dried with purified nitrogen gas.

To measure the effects of the solvent (hexane) application, we performed a second assay, again involving two groups. For the first group, nymphs were rinsed with 100 μl hexane for 10 min. The hexane in each vial was then evaporated with purified nitrogen gas, so the cuticular hydrocarbons could re-adhere to the outer surface of the insects. For the second group, nymphs were rinsed in 100 μl hexane for 10 min to remove their cuticular hydrocarbons and then removed from the solvent and dried with purified nitrogen gas^14^.

To test if surface chemicals from *F*. *racemosa* inflorescence branches elicit similar behaviors from ants as do *Tricentrus* sp. cuticular hydrocarbons, we performed a third assay. For one group, the cuticular hydrocarbons of *Tricentrus* sp. nymph were removed as previously described; for the second group, cuticular hydrocarbons of *Tricentrus* sp. nymphs were again removed, but replaced with surface chemicals from *F*. *racemosa* branches. These *F*. *racemosa* surface chemicals were obtained by first rinsing in 4 ml hexane for 10 min sequentially five small inflorescence branches. Three randomly selected *Tricentrus* sp. nymphs were then rinsed with the resulting solution, and the solvent removed by drying with purified nitrogen gas^14^.

For the behavioural assays, each dead nymph was stuck to a 5 mm × 5 mm square filter paper using 0.5 μl of liquid glue, which was left to dry for 15 min. One large branch on a mature *F*. *racemosa* tree was selected and two areas were specified on the branch. Two papers, one for each nymph group, were pinned to each area. Test areas were reversed between treatment groups between repeats. Each assay lasted for up to 15 min. Within this time period, we recorded which of the two *Tricentrus* sp. was removed first by the ants^14,15,34^.

### The effects of ant-treehopper mutualists on the fig wasp community

We conducted a field experiment to test the effects of the ant-treehopper mutualism on the fig wasp community of *F*. *racemosa*. We recorded ant and treehopper abundances for each fruit branch, then categorized them into four levels of ant-treehopper abundance: (1) an ant-free treatment for which no ants and treehoppers were observed during the period (5 weeks); (2) a ‘few ants’ treatment, for which about 6.17 ± 3.76 (mean ± SD) ants and 1.00 ± 0.63 (mean ± SD) treehoppers were observed during the period, and (3) a ‘many ants’ treatment, for which about 20.20 ± 5.07 (mean ± SD) ants and 5.80 ± 1.64 (mean ± SD) treehoppers were observed. (4) To test the absolute effect of ant-treehopper presence on the fig-fig wasp mutualism, we also used a fourth treatment, which excluded ants by the use of flavorless, colorless rat-stop glue (Yiwu Xinqi Super Glue Product Factory, Zhejiang, China)^4^ and all treehoppers were physically removed at the start of the experiment.

After approximately four weeks, the near-mature figs were collected and put into fine mesh bags for 24 h to collect the dispersing wasps. All wasps were then killed and stored at −20 °C. All wasps were sorted by species and sex, identified to species and counted. After wasp emergence, each fig was cut longitudinally into four equal parts. Seeds from one quarter (randomly chosen) were then counted. The total number of seeds per fig was estimated by multiplying the quarter segment count by four^4^.

### Data analyses

The relationship between *Tricentrus* and *O*. *smaragdina* abundances were quantified with Pearson’s correlation.

Cluster analysis was used to classify chemical profiles of each sample into different groups based on Horn–Morisita distances. The clustering strategy used was the un-weighted pair-group method, using arithmetic averages (UPGMA). We then used non-metric multidimensional scaling (NMDS) to visualize chemical profile differences (Horn-Morisita distance) in two dimensions. Before analysis, we performed a square-root transformation and then used Wisconsin double standardization to normalize the data.

In the attractiveness of chemicals to ants experiment, we used a generalized linear mixed model (GLMM), with ‘tree’ as random factor, ‘chemical treatment’ as a fixed factor, and the number of ants as the response. Ant number data were modeled using a Poisson distribution family using the log link function. The pairwise comparisons between ‘chemical treatment’ using Tukey’s HSD (honest significant difference).

For the dual choice behavioral assays, chi-square tests were used to test the difference in the frequencies of *Tricentrus* removed by the ants. The null hypothesis was that ants were equally likely to remove *Tricentrus* regardless of treatment.

We used analysis of similarity (ANOSIM) to examine how ant-treehopper abundances affect wasp community structure between different levels of ant-treehopper abundance based on Bray–Curtis distances and each test ran for 999 permutations. The effects of ant-treehopper abundances on the abundances of six species of fig wasps and seeds were quantified with generalized linear models. For each model, ant-treehopper abundance levels were fixed factors. ‘Tree’ was included in each model as a random factor to control for any effects of unmeasured variation among trees. Interactions between explanatory variables were not included in any model. Multiple pair-wise-comparisons between different levels of ant-treehopper abundances were performed using the Student-Newman-Keuls method. All analyses were conducted with R 3.0.3^35^.

### Data and materials availability

All data needed to evaluate the conclusions in the paper are present in the paper and/or the Supplementary Materials (10.6084/m9.figshare.1025771). Additional data related to this paper may be requested from the authors.

## Electronic supplementary material

supplementary dataset 1
