# Supplementary material for: Chemical camouflage: a key process in shaping an ant-treehopper and fig-fig wasp mutualistic network
Source: Sci Rep. 2018 Jan 30;8:1833. doi: 10.1038/s41598-018-20310-7 (PMC5789893; doi:10.1038/s41598-018-20310-7)
Supplement: Supplementary file 1 — supplementary dataset 1 [file 41598_2018_20310_MOESM1_ESM.doc]

**Supplementary information for manuscript " Chemical camouflage: a key process in shaping an ant-treehopper and fig-fig wasp** **mutualistic network"**

Bo Wang, Min Lu, James, M. Cook, Da-Rong Yang, Derek W. Dunn, Rui-Wu Wang

Table 1. Occurrence and relative amounts of cuticular hydrocarbon (mean±SE) from, *Tricentrus* sp., *Oecophylla smaragdina*, *Ceratosolen fusciceps* and *Platyneura mayri*, and surface chemicals of *Ficus racemosa* branches.

| **Compounds** | | | | **RI** | | **Species sampled** | | | | | | | | | |
| --- | --- | --- | --- | --- | --- | --- | --- | --- | --- | --- | --- | --- | --- | --- | --- |
|  |  |  |  |  |  | ***F. racemosa*** | | ***Tricentrus* sp.** | | ***O. smaragdina*** | | ***C. fusciceps*** | | ***P. mayri*** | |
| **Alcohol** | | | | | | | | | | | | | | | |
| Elemol | | | | 1548.29 | | ND | | ND | | 3.55±0.5 | | 0.24 | | ND | |
| Labd-7, 13-dien-15-ol | | | | 2291.79 | | ND | | ND | | ND | | ND | | 22.98±0.91 | |
| 3-Hydroxy-Manool | | | | 2293.61 | | ND | | ND | | 0.8 | | ND | | 0.9±0.03 | |
| 1-Eicosanol | | | | 2527.59 | | ND | | ND | | ND | | 1.97 | | 5.92±0.24 | |
| Sugiol | | | | 2631.44 | | 0.56±0.08 | | 7.11±0.9 | | ND | | ND | | ND | |
| 1,30-Triacontanediol | | | | 3170.32 | | 3.5±0.44 | | 27.3±3.34 | | ND | | ND | | ND | |
| Alpha-Amyrin* | | | | 3234.74 | | 4.85±0.39 | | ND | | ND | | ND | | ND | |
| Lupenol* | | | | 3260.3 | | 6.22±0.59 | | ND | | ND | | ND | | ND | |
| **Alkane** | | | | | | | | | | | | | | | |
| Heptacosane | | | | 2758.26 | | 0.49±0.19 | | 3.43±0.28 | | ND | | ND | | 0.84±0.02 | |
| Nonacosane | | | | 2905.71 | | ND | | ND | | 4.7±1.63 | | ND | | 1.07±0.15 | |
| Octadecane, 3-Ethyl-5-(2-Ethylbutyl)* | | | | 2958.82 | | ND | | ND | | 2.7±0.81 | | 5.37±0.77 | | 0.99±0.07 | |
| Hexatriacontane* | | | | 2994.3 | | 2.75±0.3 | | 2.31±0.09 | | ND | | 9.6±1.53 | | 0.88±0.08 | |
| Triacontane | | | | 3006.39 | | ND | | 2.23±0.51 | | ND | | 1.55±0.35 | | ND | |
| Untriacontane | | | | 3099.61 | | ND | | ND | | ND | | 1.59 | | 7.24±1.64 | |
| Dotriacontane | | | | 3217.85 | | 12.02±1.06 | | 19.36±2.82 | | ND | | 9.62±1.5 | | ND | |
| Tetratetracontane* | | | | 3439.62 | | ND | | ND | | ND | | 5.19±0.71 | | 2.43±0.45 | |
| Hentriacontane^#^ | | | |  | | ND | | 1.41 | | 2.19±0.5 | | ND | | 1.88±0.43 | |
| Tritetracontane^#^ | | | |  | | ND | | 4.16 | | ND | | 5.45±0.87 | | ND | |
| Tetracontane^#^ | | | |  | | ND | | ND | | ND | | 1.57 | | 4.52±0.92 | |
| Pentatriacontane^#^ | | | |  | | ND | | ND | | ND | | 1.87±0.34 | | 4.52±0.43 | |
| **Aldehyde** | | | | | | | | | | | | | | | |
| Cuparenal | | | | 1749.79 | | ND | | ND | | 4.7±0.52 | | 2.48±0.34 | | ND | |
| Ocadecanal | | | | 2446.82 | | 6.63±0.78 | | 5.28±0.28 | | ND | | ND | | ND | |
| **Alkene** | | | | | | | | | | | | | | | |
| Camphene Hydrate | | | | 1145.73 | | ND | | ND | | 27.91±2.52 | | ND | | ND | |
| α-Cubebene | | | | 1348.08 | | ND | | ND | | 1.27±0.39 | | ND | | ND | |
| 14-hydroxy-Cadinene | | | | 1804.43 | | ND | | 7.98±0.15 | | 3.05±1.47 | | 31.37±4.6 | | 1.04±0.18 | |
| Pimaradiene | | | | 1952.42 | | ND | | ND | | 4.28±0.98 | | 2.2±0.27 | | ND | |
| Osthole | | | | 2139.08 | | ND | | ND | | ND | | 14.05±2.01 | | ND | |
| Incensole | | | | 2153.61 | | ND | | ND | | 20.21±1.99 | | ND | | ND | |
| Totarolone | | | | 2526.7 | | ND | | ND | | ND | | 1.83±0.22 | | ND | |
| Hinokienone | | | | 2528.05 | | ND | | 1.94±0.29 | | ND | | ND | | 0.85±0.03 | |
| 1-Docosene | | | | 2560.69 | | ND | | ND | | ND | | 3.2±0.15 | | 7.47±0.18 | |
| Hinokiol | | | | 2582.24 | | 0.42±0.05 | | ND | | ND | | ND | | ND | |
| 17-Pentatriacontene* | | | | 3137.63 | | 1.6±0.23 | | 1.74±0.28 | | ND | | ND | | ND | |
| **Carboxylic acid** | | | | | | | | | | | | | | | |
| Communic Acid | | | | 2364.78 | | 1.98±0.19 | | ND | | ND | | ND | | ND | |
| 11,14,17-Eicosatrienoic Acid | | | | 2530.49 | | ND | | ND | | ND | | ND | | 27.87±1.57 | |
| Hexacosanoic Acid | | | | 2823.08 | | 3.41±0.7 | | ND | | ND | | ND | | ND | |
| Dotriacontane | | | | 3195.95 | | 1.32±0.18 | | ND | | ND | | ND | | ND | |
| Olean-12-En-3-yl Acetate^*^ | | | | 3426.38 | | 3.75±0.31 | | ND | | ND | | ND | | ND | |
| **Ester** |  |  | |  | |  | |  | |  | |  | |  | |
| Ethyl Nerolate | | | | 1351.23 | | ND | | ND | | 4.09±0.47 | | ND | | ND | |
| Methyl Sandaracopimarate | | | | 2252.53 | | ND | | ND | | 15.42±2.03 | | ND | | ND | |
| Methyl-7,13,15-Abietatrienoate | | | | 2522.93 | | ND | | ND | | 1.69 | | ND | | 1.59±0.15 | |
| Lupenyl Acetate | | | | 3460.1 | | 16.86±1.95 | | ND | | ND | | ND | | ND | |
| **Ether** |  |  |  | |  | |  | |  | |  | |  | |  |
| Octacosanol Trimethylsilyl Ether | | | | 3184.02 | | 10.87±1.87 | | 6.15±0.41 | | ND | | ND | | 2.33±0.52 | |
| **Ketone** |  | | | | | | | | | | | | | |  |
| 2-Tetradecanone | | | | 2622.4 | | 0.52±0.06 | | ND | | 2.53±0.93 | | ND | | 2.09±0.31 | |
| **Olefine Ketone** | | | | | | | | | | | | | | | |
| Lupenone^*^ | | | | 3244.67 | | 21.52±2.89 | | ND | | ND | | ND | | ND | |
| **Phenol** | | | | | | | | | | | | | | |  |
| Ferruginol | | | | 2370.51 | | ND | | ND | | ND | | 1.77±0.24 | | 1.75±0.03 | |
| Methylenebis^*^ | | | | 3345.97 | | ND | | 20.45±4.96 | | 5.28 | | ND | | ND | |

Notes: ND= Not detected. ^*^ No corresponding arithmetic index. Here give the chemical with similar mass spectrua. ^#^Chemicals with carbon number over 40.
